# Robotic pancreaticoduodenectomy provides better histopathological outcomes as compared to its open counterpart: a meta-analysis

**DOI:** 10.1038/s41598-021-83391-x

**Published:** 2021-02-12

**Authors:** Xiang Da Dong, Daniel Moritz Felsenreich, Shekhar Gogna, Aram Rojas, Ethan Zhang, Michael Dong, Asad Azim, Mahir Gachabayov

**Affiliations:** 1 Department of Surgery, Westchester Medical Center, New York Medical College, Valhalla, NY USA; 2 Division of General Surgery, Department of Surgery, Vienna Medical University, Vienna, Austria; 3 Taylor Pavilion, Suite D-365, 100 Woods Road, Valhalla, NY 10595 USA; 4 Taylor Pavilion, Suite D-361, 100 Woods Road, Valhalla, NY 10595 USA

**Keywords:** Surgical oncology, Outcomes research, Pancreatic cancer, Pancreas, Pancreatic disease

## Abstract

The aim of this meta-analysis was to evaluate whether robotic pancreaticoduodenectomy (PD) may provide better clinical and pathologic outcomes compared to its open counterpart. The Pubmed, EMBASE, and Cochrane Library were systematically searched. Overall postoperative morbidity and resection margin involvement rate were the primary endpoints. Secondary endpoints included operating time, estimated blood loss (EBL), incisional surgical site infection (SSI) rate, length of hospital stay (LOS), and number of lymph nodes harvested. Twenty-four studies totaling 12,579 patients (2,175 robotic PD and 10,404 open PD were included. Overall postoperative mortality did not significantly differ [OR (95%CI) = 0.86 (0.74, 1.01); p = 0.06]. Resection margin involvement rate was significantly lower in robotic PD [15.6% vs. 19.9%; OR (95%CI) = 0.64 (0.41, 1.00); p = 0.05; NNT = 23]. Operating time was significantly longer in robotic PD [MD (95%CI) = 75.17 (48.05, 102.28); p < 0.00001]. EBL was significantly decreased in robotic PD [MD (95%CI) = − 191.35 (− 238.12, − 144.59); p < 0.00001]. Number of lymph nodes harvested was significantly higher in robotic PD [MD (95%CI) = 2.88 (1.12, 4.65); p = 0.001]. This meta-analysis found that robotic PD provides better histopathological outcomes as compared to open PD at the cost of longer operating time. Furthermore, robotic PD did not have any detrimental impact on clinical outcomes, with lower wound infection rates.

## Introduction

Pancreatic surgery has made tremendous progress over the last several decades. With the introduction of minimally invasive techniques, adoption of laparoscopy and robotic platforms for performance of complex pancreatic surgery has evolved as well. Pancreaticoduodenectomy (PD) remains one of the most technically challenging surgeries in the current era^1–3^. Prior to the introduction of the robotic platform, attempts to improve surgical outcome with the use of laparoscopy did not gain widespread adoption^4,5^. Introduction of robotic surgery has overcome some of the limitations from laparoscopic approach^6^. Difficulties related to laparoscopic surgery such as the fulcrum effect, fine instrument manipulation, and reversal of instrument tip from surgical hand-motion created challenges for many surgeons^4,5^.

Following the first case of robotic PD reported in 2003 by Giulianotti et al., multiple studies have reported feasibility and safety of robotic PD in the management of pancreatic head malignancies^5,7–11^. Robotic platform allows performance of complex surgical cases by overcoming the limitations associated with laparoscopic surgery. The advantages of robotic surgery includes 7 degrees of freedom, 3D visualization, and fine tremor reduction^3^. Previous reviews and meta-analyses have found perioperative outcomes of robotic PD to be at worst equivalent to those of open PD^12,13^. The studies to date have focused on clinical short-term outcomes in patients undergoing robotic PD. However, long-term outcomes of robotic surgery for pancreatic cancer is still scarce in the literature due to the limited longitudinal data available.

Based on longitudinal studies, we know that margin negative (R0) resection for pancreatic cancer translates into improved survival^14–16^. In addition, increased number of lymph nodes retrieved during surgery frequently allows accurate staging and is synonymous with the adequacy of surgical resection^17^. There are currently several studies that also reports improved resection margin with the use of robotic platforms^12^. Based on the available literature, we attempted to evaluate the role of robotic PD in comparison to open approach with a focus on short-term clinical outcomes as well as histopathological outcomes such as margin status and nodal harvesting^14^.

## Materials and methods

This systematic review was performed according to the Cochrane Handbook for Systematic Reviews of Interventions^18^ and follows the Preferred Reporting Items for Systematic Reviews and Meta-Analyses (PRISMA) and Meta-analysis Of Observational Studies in Epidemiology (MOOSE) guidelines^19,20^. The protocol of this systematic review was developed prospectively and registered in the International prospective register of systematic reviews PROSPERO: CRD42018112039. Given the summary design nature of this study, Institutional Review Board approval and written consents were not required. The literature search, screening of the records, study selection, extraction and analysis of the data, followed by critical appraisal, were performed by two independent researchers (MG and XDD). The research question was formulated within the PICOTS framework as following:

(P) Population: Adults older than 18 years old undergoing pancreaticoduodenectomy.

(I) Intervention: robotic pancreaticoduodenectomy.

(C) Comparator intervention: open pancreaticoduodenectomy.

(O) Outcomes: operating time, estimated blood lost, postoperative complication rate, postoperative pancreatic fistula (POPF) rate, delayed gastric emptying rate, incisional surgical site infection rate, reoperation rate, length of hospital stay, margin involvement rate, and number of lymph nodes harvested.

(T) Time: Short-term.

(S) Setting: Inpatient.

### Eligibility criteria, definitions and endpoints

All experimental or observational clinical studies comparing robotic to open PD for benign and/or malignant disease were eligible for inclusion. Non-comparative descriptive studies, studies comparing any of the interventions of interest to a non-relevant intervention such as laparoscopic pancreaticoduodenectomy or robotic tumor resection followed by mini-laparotomy for reconstruction, and review articles were excluded.

Postoperative complications were classified according to Clavien-Dindo classification^21^. Surgical site infections (SSI) were defined according to the Center for Disease Control National Nosocomial Infections Surveillance System^22^.

The primary endpoints of this systematic review were overall postoperative complication and resection margin involvement rates. Secondary endpoints included operating time, estimated blood loss, postoperative complication rate, postoperative pancreatic fistula rate, rate of delayed gastric emptying, surgical site infection rate, reoperation rate, length of hospital stay, and number of lymph nodes harvested.

### Search strategy and study selection

The Pubmed, EMBASE, and Cochrane Library were systematically searched using the following MeSH terms: ‘pancreatoduodenectomy’, ‘pancreaticoduodenectomy’, ‘whipple’, and ‘robotic’ combined with the Boolean operator ‘AND’ and all synonyms combined with the Boolean operator ‘OR’. In addition, clinicaltrials.gov was searched for any ongoing studies. The details of Pubmed search strategy are presented in Supplement 1. Relevant articles were identified, and the results of the search were screened through the title, abstract and/or full text article. The sensitivity of the search strategy was tested by screening the references of included articles for additional publications.

### Data extraction and quality assessment

The data from the included articles were collected to predefined Microsoft Excel tables and studies were assessed for validity by three researchers independently (MG, XDD, and DMF). Extracted data items included publication-specific variables (authors and affiliations, journal and year of publication), study-specific variables (study design, study span, sample size, definitions of interventions and endpoints, conclusions, potential biases), and patient-specific variables (baseline characteristics, intra- and postoperative outcomes, pathologic outcomes). Quality assessment of each individual study was performed according to Cochrane Handbook for Systematic Reviews of Interventions on the following items: selection, performance, detection, attrition, selective reporting, and other bias risks^18^. In addition, Risk Of Bias In Non-randomized Studies (ROBINS-I) tool was utilized to evaluate the quality of observational studies on the following biases: confounding, selection, classification of interventions, deviations of intended comparability, and outcomes^23^.

### Statistical analysis

Inverse variance method with mean difference (MD) and standard error as the measure of an effect estimate was used for continuous variables, whereas Mantel–Haenszel method with odds ratios and 95% confidence intervals (OR (95%CI)) was employed for dichotomous variables. In cases when continuous variables were reported in median and interquartile range in the included studies, mean and standard deviation were estimated using Hozo’s formula^24^. Statistical heterogeneity among effect estimates was assessed using Cochran Chi^2^ and I^2^, and between-study variance was assessed using Tau^2^ statistic when the I^2^ was 50% or greater^25^. Random-effects model was utilized for meta-analysis. The results of the meta-analysis were illustrated on forest plots. Ad-hoc meta-regression analysis with Omnibus test was performed to evaluate the impact of potential confounding factors on outcomes. To assess clinical significance of the statistical findings for dichotomous endpoints, relative risk reduction (RRR), absolute risk reduction (ARR) and number needed to treat/harm (NNT) with 95%CI were calculated. Clinical significance of the MD was assessed for numeric endpoints. The variability of the effect of intervention over different settings was assessed using 95% prediction intervals^26^. Visual assessment of funnel plots and Egger’s test were utilized to assess for publication bias. A leave-one-out meta-analysis was utilized for sensitivity analysis. A p-value < 0.05 was considered statistically significant. Certainty of evidence was evaluated using GRADE approach. Statistical analysis was performed using RevMan (version 5.3; Nordic Cochrane Center, Cochrane Collaboration, Copenhagen, Denmark) and CMA Software (Version 3; Biostat, NJ, USA).

## Results

### Literature search and study selection

**Figure 1:**
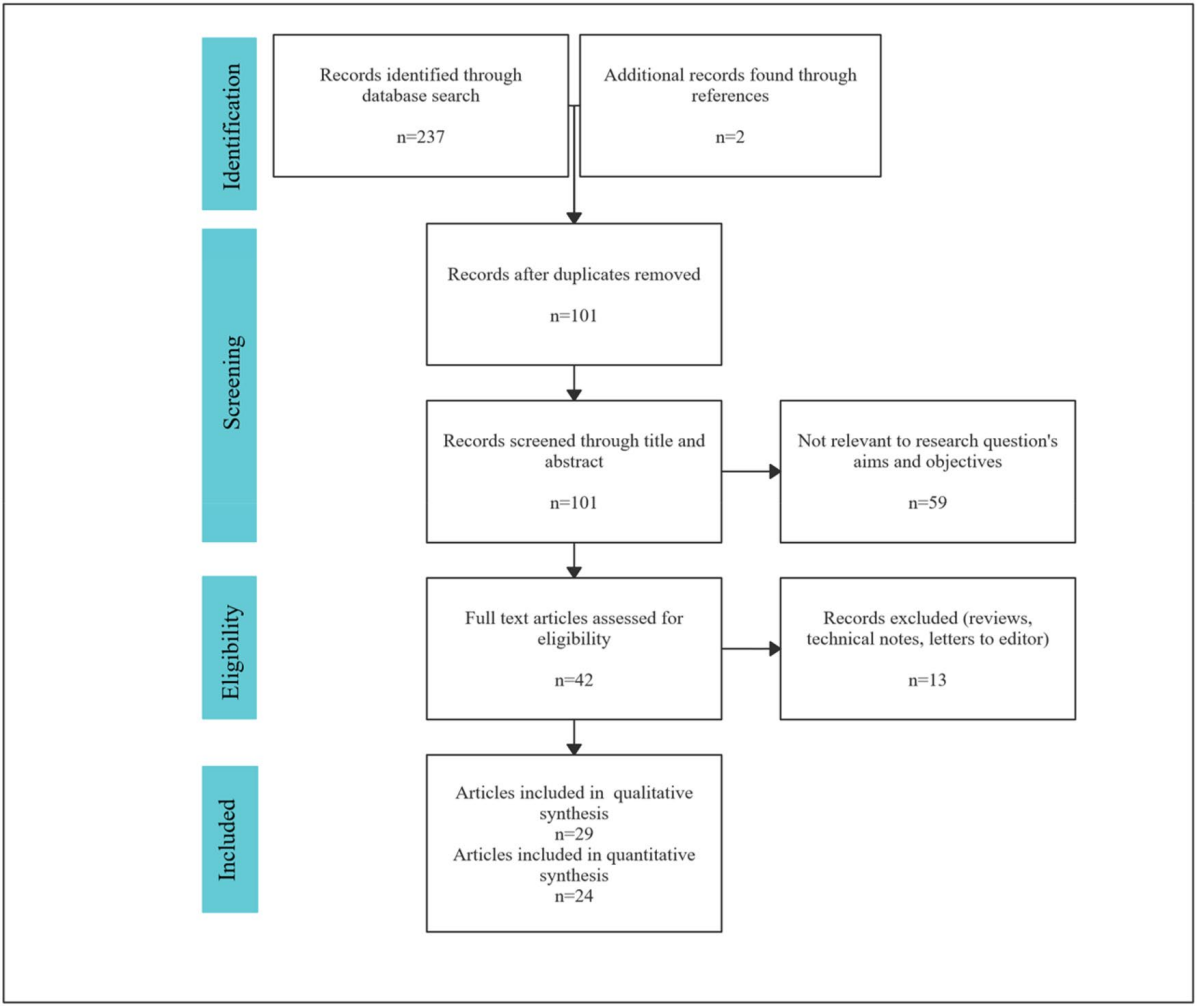
PRISMA flow diagram.

Details of the search strategy and study selection are presented in the PRISMA flowchart (Fig. 1). Four databases were searched and revealed 237 records. Additionally, two articles were found at clinicaltrials.gov and through the references of eligible studies. Twenty-nine studies (including published abstracts of conference proceedings) were included in the qualitative synthesis after excluding duplicates, non-relevant articles, and articles not reporting the outcome of interest.

### Quality assessment

**Figure 2:**
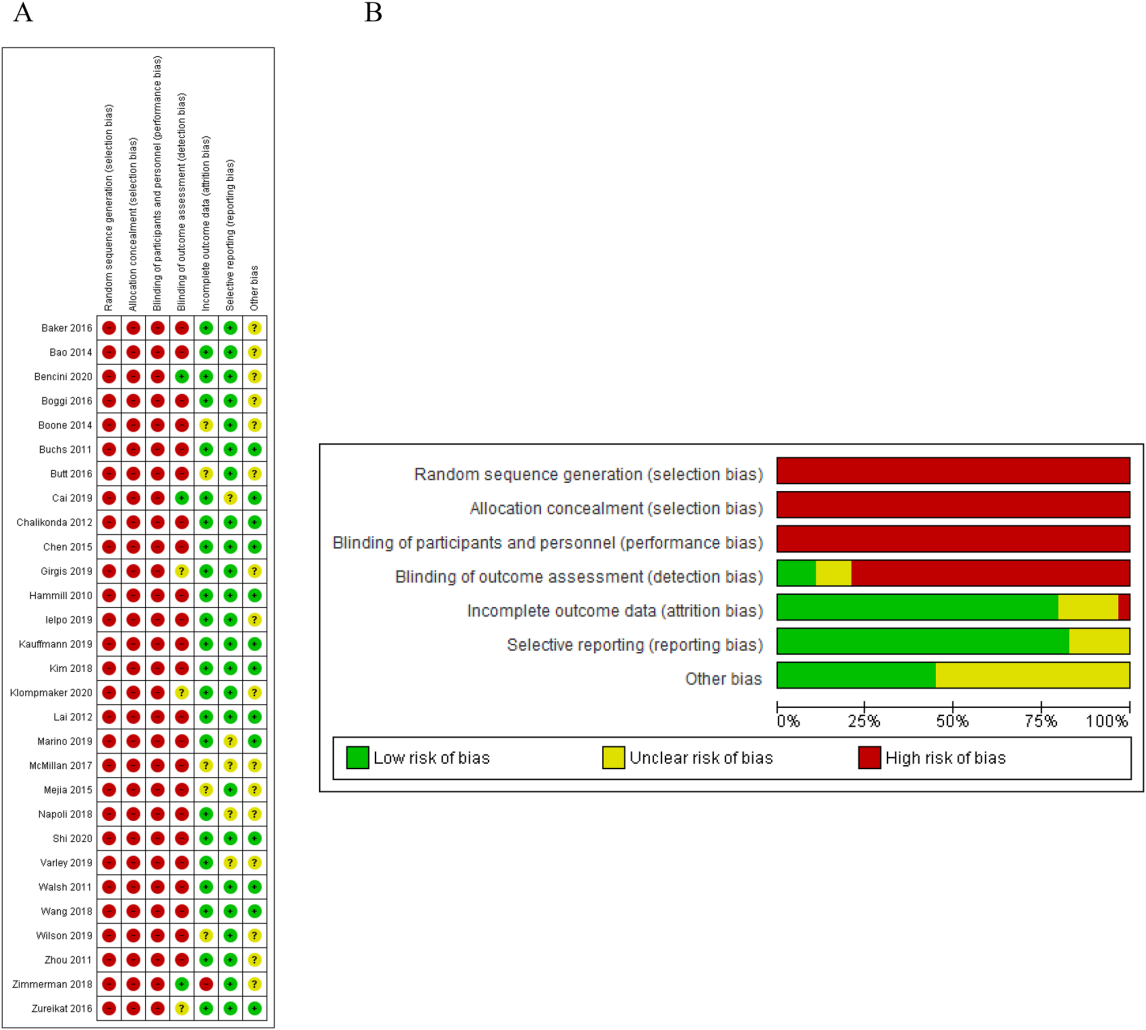
Quality assessment: (**A**) Risk of bias summary. (**B**) Risk of bias graph.

The risk of bias summary and graph of the included studies are presented in Fig. 2A,B. The risk of selection, performance and detection bias was high in all included studies given their observational nature. Attrition, reporting, and other bias risks were moderate or low in included studies. The results of quality assessment using the ROBINS-I tool are presented in Supplement 2. Overall risk of bias was assessed as serious in most studies.

### Description of included studies

**Figure 3:**
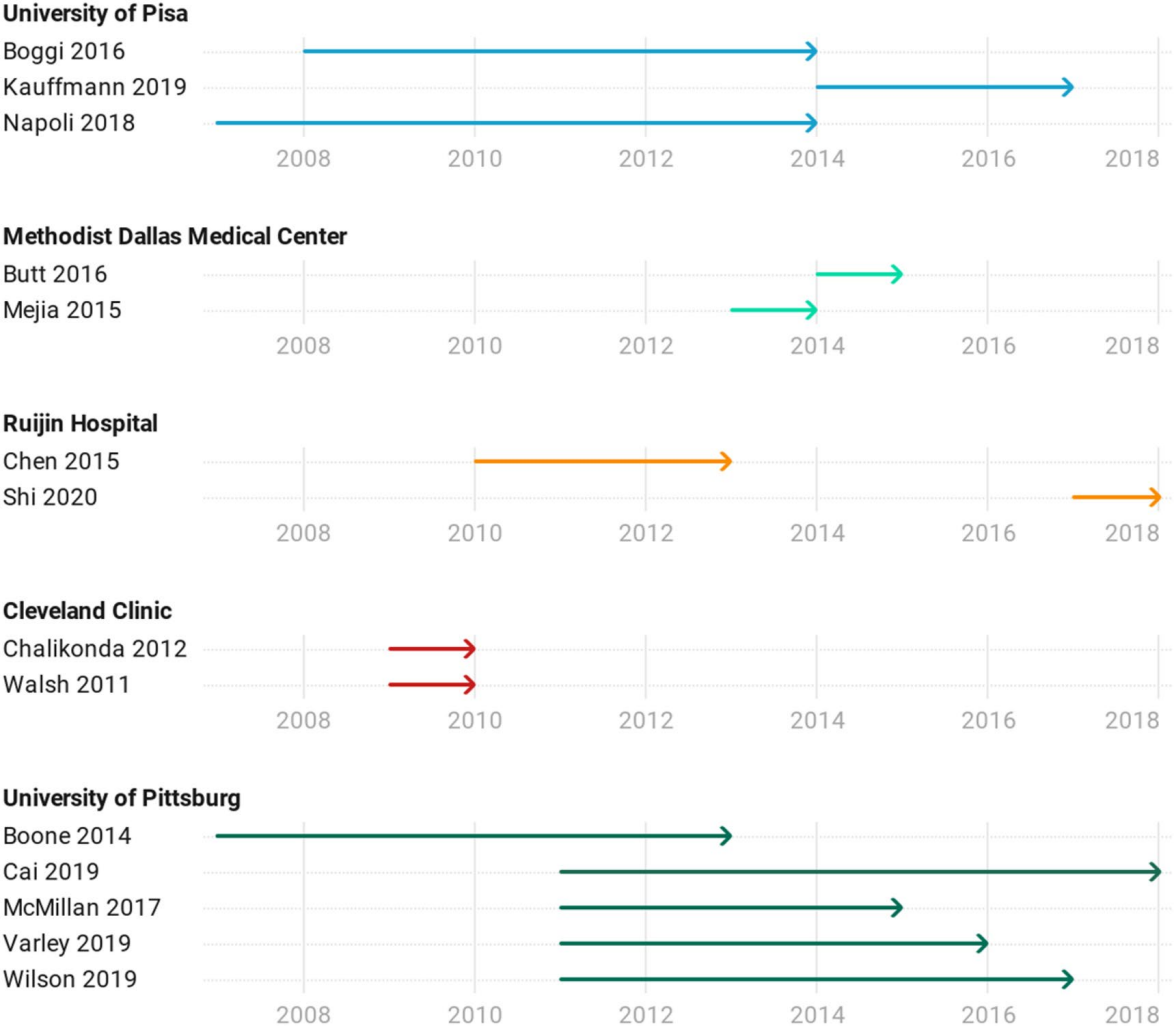
Arrow plot of study spans of included studies with the risk of duplicate data synthesis.

Figure 3 highlights the time span of included studies published from the same institutions, which may increase the risk of duplicate data synthesis. Due to an overlap of the studies by Napoli et al.^27^ and Boogi et al.^28^ from the University of Pisa, only the study by Boogi et al.^28^ was included as it covers a longer time span. An abstract published by Walsh et al.^29^ from Cleveland Clinic was excluded as there was an overlap with the study by Chalikonda et al.^30^ There were five studies from the University of Pittsburgh that overlap to a certain extent. After excluding three (McMillan et al.^31^, Varley et al.^32^ and Wilson et al.^33^), studies by Boone et al.^34^ and Cai et al.^35^ with a maximal time span covered and minimal overlap were included.

Twenty-four studies were included in the final quantitative data synthesis, totaling 12,579 patients (2,175 robotic PD and 10,404 open PD)^8,28,30,34–54^. Seven studies^30,34,36,38,41,50,52^ were prospective cohort studies and 17 retrospective cohort studies^8,28,35,37,39,40,42–49,51,53,54^. In four of these studies^34,40,43,50^, data were extracted from the abstracts of conference proceedings published in indexed journals. Five studies^8,36,38,41,52^ had the Oxford CEBM level of evidence of 2b and 19 studies-2c^28,30,35,37,39,40,42–51,53–55^. Three studies^8,42,47^ were multicenter studies with (5, 8 and 14 included centers), 20 studies^28,30,35–41,43–46,48–54^ were single center studies and one study^34^ did not provide this information. Various primary endpoints were reported in seven studies^35,38,45,47,50,52,54^ and are described in Table 1, whereas 17 studies^8,28,30,34,36,37,39–44,46,48,49,51,53^ did not report/specify this variable.

**Table 1:** Characteristics of included studies.

Author	Publication	Design	Number of centers involved	Primary endpoint(s)	Sample size (total n = 12,579)	Number of patients (Robotic vs. Open) (total 2,175 vs. 10,404)	Indication for surgery (benign or malignant disease)	Primarily involved organ (pancreas, biliary tract, duodenum)	Level of evidence (Oxford CEBM)
Baker	Int J Med Robot 2016^36^	Prospective cohort study (2012–2013)	1	NS	71	22 vs. 49	B + M	P + BT + D	2b
Bao	J Gastrointest Surg 2014^37^	Retrospective cohort study (2009–2011)	1	NS	56	28 vs. 28	B + M	P + BT + D	2c
Bencini	Surg Endosc 2020^38^	Prospective cohort study (2014–2018)	1	Postoperative (30-day) events	121	38 vs. 83	B + M	P + BT + D	2b
Boggi	World J Surg 2016^28^	Retrospective cohort study (2008–2014)	1	NS	119	83 vs. 36	B + M	P + BT + D	2c
Boone	HPB 2014^34^	Abstract; Prospective cohort study (2008–2013)	NR	NS	156	58 vs. 98	NR	NR	2c
Buchs	World J Surg 2011^39^	Retrospective cohort study (2002–2010)	1	NS	83	44 vs. 39	B + M	P + BT + D	2c
Butt	HPB 2016^40^	Abstract; Retrospective cohort study (2014–2015)	1	NS	67	12 vs. 55	NR	NR	2c
Cai	J Gastrointest Surg 2019^35^	Retrospective cohort study (2011–2018)	1	CR-POPF rate	865	460 vs. 405	B + M	NR	2c
Chalikonda	Surg Endosc 2012^30^	Prospective cohort study (2009–2010)	1	NR	60	30 vs. 30	B + M	NR	2c
Chen	Surg Endosc 2015^41^	Prospective cohort study (2010–2013)	1	NR	180	60 vs. 120	B + M	P + BT + D	2b
Girgis	Ann Surg 2019^42^	Retrospective cohort study (2011–2016)	5	NR	361	163 vs. 198	M	P + BT + D	2c
Hammill	HPB 2010^43^	Abstract; Retrospective cohort study (2005–2009)	1	NR	77	8 vs. 69	B + M	P + BT	2c
Ielpo	Updates Surg 2019^44^	Retrospective cohort study (2010–2017)	1	NS	34	17 vs. 17	B + M	NR	2c
Kauffmann	Surg Endosc 2019^45^	Retrospective cohort study (2014–2017)	1	Positive margin rate	268	93 vs. 175	M	P	2c
Kim	J Hepatobiliary Pancreat Sci 2018^46^	Retrospective cohort study (2015–2017)	1	NR	237	51 vs. 186	B + M	P + BT + D	2c
Klompmaker	Ann Surg 2020^47^	Retrospective cohort study (2012–2017)	14	30-day morbidity	920	191 vs. 729	B + M	P + BT + D	2c
Lai	Int J Surg 2012^48^	Retrospective cohort study (2000–2012)	1	NS	87	20 vs. 67	B + M	P + BT + D	2c
Marino	J Robot Surg 2019^49^	Retrospective cohort study (2014–2016)	1	NR	70	35 vs. 35	M	P + BT + D	2c
McMillan^§^	Jama Surg 2017^31^	Retrospective cohort study (2003–2015)	16	POPF rate	2,846	185 vs. 2661	B + M	P + BT + D	2c
Mejia	Surg Endosc 2015^50^	Abstract; Prospective cohort study (2013–2014)	1	Morbidity	26	14 vs. 12	NR	NR	2c
Napoli^§^	Surg Endosc 2018^27^	Retrospective cohort study (2007–2014)	1	CR-POPF rate	309	82 vs. 227	B + M	P + BT + D	2c
Shi	JAMA Surg 2020^51^	Retrospective cohort study (2017–2018)	1	NR	834	200 vs. 634	B + M	P + BT + D	2c
Varley^§^	HPB 2019^32^	Retrospective cohort study (2011–2016)	1	Length of hospital stay	282	133 vs. 149	B + M	P + BT + D	2c
Walsh^§^	Surg Endosc 2011^29^	Abstract; Retrospective cohort study (2009–2010)	1	NR	50	25 vs. 25	NR	NR	2c
Wang	Surgery 2018^52^	Prospective cohort study (2012–2017)	1	CR-POPF rate	296	118 vs. 178	B + M	P + BT + D	2b
Wilson^§^	HPB 2019^33^	Abstract; Retrospective cohort study (2011–2017)	1	NR	190	116 vs. 74	M	P + BT + D	2c
Zhou	Int J Med Robot 2011^53^	Retrospective cohort study (2009)	1	NR	16	8 vs. 8	M	P + BT + D	2c
Zimmerman	HPB 2018^54^	Retrospective cohort study (2014–2015)	1	30-day mortality and morbidity	6547	211 vs. 6,336	B + M	P + BT + D	2c
Zureikat	Ann Surg 2016^8^	Retrospective cohort study (2011–2015)	8	NS	1028	211 vs. 817	B + M	P + BT + D	2b

*CEBM* Centers for Evidence-Based Medicine; *B* benign; *M* malignant; *P* pancreas; *BT* biliary tract; *D* duodenum; *NR* not reported; *NS* not specified.

^§^Studies that were excluded from the quantitative synthesis.

### Description of study populations and interventions

Adult patients from multiple countries (China, Korea, Italy, USA, Russia, Netherlands, Belgium, France, Turkey, Germany, UK and Spain) were involved in the 24 included studies. 17 studies^8,28,30,35–39,41,43,44,46–48,51,52,54^ included patients with benign and malignant diseases, four studies^42,45,49,53^ only malignant diseases and three studies^34,40,50^ did not describe the indications. The primarily involved organs were the pancreas, biliary tract and duodenum in 16 studies^8,28,36–39,41,42,46–49,51–54^; one study^43^ only involved only pancreatic and biliary tract diseases and one study^45^ only pancreatic cancer. Six studies^30,34,35,40,44,50^ did not provide this information (Table 1).

Patients’ baseline characteristics are summarized in Table 2. The definitions of the interventions are summarized in Table 3. Robotic PD was a totally robotic procedure in 17 studies^8,28,34–36,38,39,41,42,44,45,47,49,51–54^. A hybrid procedure was performed in four studies^30,37,46,48^ and three studies^40,43,50^ did not specify the type of the procedure. Six studies reported DaVinci console type (both Si/Xi in three studies^38,44,52^; Si^49,50^ in two and S in one^41^).

**Table 2:** Comparison of patients’ baseline characteristics in robotic vs. open pancreaticoduodenectomy.

Included studies	Age (years)		Gender (% male)		BMI (kg/m^2^)		ASA > 2	
	Robotic	Open	Robotic	Open	Robotic	Open	Robotic	Open
Baker 2016^36^	63 (38–82)*	63 (26–86)*	5%	63%	26 (18–35)*	27 (16–38)*	68%	82%
Bao 2014^37^	68 ± 11.2	67.7 ± 12.5	46%	46%	26 (19–40)*	26 (19–40)*	NR	NR
Bencini 2020^38^	60 (42–73)*	74 (56–91)*	58%	53%	26 (18–32)*	24 (14–38)*	16%	36%
Boggi 2016^28^	62 (50–71)*	64 (56–74)*	45%	53%	24 (23–24)*	23 (22–25)*	33%	36%
Boone 2014^34^	NR	NR	NR	NR	NR	NR	NR	NR
Buchs 2011^39^	63 ± 14.5	56 ± 15.8	50%	36%	27.7 ± 5.4	24.8 ± 4.7	NR	NR
Butt 2016^40^	NR	NR	NR	NR	NR	NR	NR	NR
Cai 2019^35^	66.5 ± 11.0	67.5 ± 10.7	55%	52%	27.8 ± 5.8	27.2 ± 5.9	NR	NR
Chalikonda 2012^30^	62	61	54%	54%	24.8	25.6	53%	76%
Chen 2015^41^	53.6 ± 13.5	53.8 ± 14.3	57%	54%	23.2 ± 2.7	22.6 ± 3.4	1.7%	1.6%
Girgis 2019^42^	66.6 ± 10.9	67.6 ± 10.3	53%	53%	27.1 ± 5.6	26.4 ± 5.3	NR	NR
Hammill 2010^43^	55	62.5	NR	NR	26.1	26.6	NR	NR
Ielpo 2019^44^	66.8 ± 9.5	61.4 ± 11.9	47%	59%	23.8 ± 4.1	24.6 ± 3.36	35%	24%
Kauffmann 2019^45^	65 (59–75)*	73 (60–79)*	50%	54%	23.1 ± 3.2	24.1 ± 3.1	NR	NR
Kim 2018^46^	60.7 ± 11.9	65.4 ± 10.1	58%	47%	22.7 ± 2.5	24.0 ± 3.1	2%	5%
Klompmaker 2020^47^	NR	34.6 ± 11.7	NR	50%	NR	24.8 ± 4.0	NR	20%
Lai 2012^48^	66.4 ± 11.9	62.1 ± 11.2	60%	57%	NR	NR	0%	0%
Marino 2019^49^	60.4 (43–72)*	62.3 (45–73)*	54%	43%	23.8 (19.4–30.9)*	23.5 (18.8–28.1)*	20%	23%
McMillan 2017 ^31 §^	NR	NR	NR	NR	NR	NR	NR	NR
Mejia 2015^50^	67.3 ± 8	62 ± 10	71%	58%	27 ± 5	27.2 ± 5	NR	NR
Napoli 2018 ^27 §^	62 (52–71)*	67 (60–75)*	44%	55%	23.5 ± 0.4	24.8 ± 0.2	42%	66%
Shi 2020^51^	59.4 ± 12.6	62.7 ± 10.5	56%	60%	NR	NR	4%	6%
Varley 2019 ^32 §^	66.3 ± 10.6	67.0 ± 10.5	48%	53%	27.5 ± 6.1	26.7 ± 5.6	89%	86%
Walsh 2011 ^29 §^	63	62	NR	NR	24	26	50%	69%
Wang 2018^52^	NR	NR	50%	57%	NR	NR	NR	NR
Wilson 2019 ^33 §^	67.3 ± 10.3	69.8 ± 10.2	NR	NR	NR	NR	NR	NR
Zhou 2011^53^	64.4 ± 9.1	59.4 ± 9.4	63%	50%	NR	NR	NR	NR
Zimmerman 2018^54^	66 (68–72)*	65 (57–72)*	52%	54%	27.3 (23.8–30.9)*	26.5 (23.2–30.2)*	NR	NR
Zureikat 2016^8^	67 (15–86)*	65 (15–93)*	55%	52%	27.5 (18.1–47.6)*	26.1 (14.7–85.5)*	NR	NR

*BMI* body mass index; *ASA* American Society of Anesthesiologists; *NR* not reported.

*Expressed in median and interquartile range.

^§^Studies that were excluded from the quantitative synthesis.

**Table 3:** Definition of interventions in included studies.

Studies	Robotic								Open					
	Technique (TR/H)	Console type (S/Si/Xi)	Type of procedure (WP/PP/MVR)	Vein resection (%)	Type of anastomosis (PJ/PG/DtM)	PJAS used (R/S)	Location of the jejunal loop (AC/RC)	Peritoneal drain used (R/S)	Type of procedure (WP/PP/MVR)	Vein resection (%)	Type of anastomosis (PJ/PG/DtM)	PJAS used (R/S)	Location of the jejunal loop (AC/RC)	Peritoneal drain used (R/S)
Baker 2016^36^	TR	NR	PP-100%	14%	PJ + DtM	NR	RC	R	WP-12%; PP-88%	14%	PJ + DtM	NR	AC	R
Bao 2014^37^	H	NR	WP & PP	NR	PJ/PG + DtM	S	AC/RC	R	WP & PP	NR	PJ/PG + DtM	S	AC/RC	R
Bencini 2020^38^	TR	Si/Xi	WP-55%; PP-45%	0%	PG + DtM	NR	NR	R	WP-30%; PP-70%; MVR-13%	24%	PJ/PG + DtM	NR	NR	R
Boggi 2016^28^	TR	NR	WP	8%	PJ + DtM	NR	NR	R	WP	11%	NR	NR	NR	R
Boone 2014^34^	TR	NR	NR	NR	NR	NR	NR	NR	NR	NR	NR	NR	NR	NR
Buchs 2011^39^	TR	NR	WP & PP	NR	PJ/PG	NR	NR	R	WP	NR	PJ	NR	NR	R
Butt 2016^40^	NR	NR	NR	NR	NR	NR	NR	NR	NR	NR	NR	NR	NR	NR
Cai 2019^35^	TR	NR	WP	15%	PJ + DtM	S	NR	R	WP	23%	PJ + DtM	S	NR	R
Chalikonda 2012^30^	H	NR	PP-100%	0%	PJ + DtM	R	AC	R	PP-100%	0%	NR	NR	NR	R
Chen 2015^41^	TR	S	WP	5%	PJ + DtM	R	RC	R	WP & PP	7%	PJ/PG + DtM	NR	NR	NR
Girgis 2019^42^	TR	NR	WP	25%	NR	NR	NR	NR	WP	38%	NR	NR	NR	NR
Hammill 2010^43^	NR	NR	NR	NR	NR	NR	NR	NR	NR	NR	NR	NR	NR	NR
Ielpo 2019^44^	TR	Si/Xi	WP	NR	PJ + DtM	NR	NR	NR	WP	NR	PJ + DtM	NR	NR	NR
Kauffmann 2019^45^	TR	NR	WP	NR	PJ + DtM	NR	NR	R	WP	NR	NR	NR	NR	R
Kim 2018^46^	H	NR	WP-4%; PP-96%	0%	PJ + DtM	R	NR	NR	WP-26%; PP-74%	7%	PJ + DtM	R	NR	NR
Klompmaker 2020^47^	TR	NR	WP-31%; PP-67%; MVR-2%	10%	NR	NR	NR	NR	WP-31%; PP-69%; MVR-3%	10%	PJ/PG	NR	NR	NR
Lai 2012^48^	H	NR	WP	NR	PJ + DtM	R	NR	R	WP-94%; PP-6%	NR	NR	NR	NR	NR
Marino 2019^49^	TR	Si	WP	NR	PJ + DtM	R	NR	R	NR	NR	NR	NR	NR	NR
McMillan 2017 ^31 §^	TR	NR	WP	NR	PJ + DtM	S	NR	R	NR	NR	PJ/PG	S	NR	S
Mejia 2015^50^	NR	Si	NR	NR	NR	NR	NR	NR	NR	NR	NR	NR	NR	NR
Napoli 2018 ^27 §^	TR	NR	WP-7%; PP-93%	9%	PJ + DtM	S	NR	R	WP-15%; PP-85%	34%	PJ + DtM	S	NR	R
Shi 2020^51^	TR	NR	WP	NR	PJ + DtM	R	RC	R	WP & PP	NR	PJ/PG + DtM	NR	NR	NR
Varley 2019 ^32 §^	TR	NR	NR	NR	NR	NR	NR	NR	NR	NR	NR	NR	NR	NR
Walsh 2011 ^29 §^	H	NR	WP	NR	NR	NR	NR	NR	WP	NR	NR	NR	NR	NR
Wang 2018^52^	TR	Si/Xi	NR	NR	Blumgart PJ	S	RC	NR	NR	NR	Blungart PJ	S	RC	NR
Wilson 2019 ^33 §^	TR	NR	NR	NR	NR	NR	NR	NR	NR	NR	NR	NR	NR	NR
Zhou 2011^53^	TR	NR	WP-63%; PP-37%	NR	PJ + DtM	NR	NR	NR	NR	NR	PJ/PG + DtM	NR	NR	NR
Zimmerman 2018^54^	TR	NR	NR	NR	NR	NR	NR	NR	NR	NR	NR	NR	NR	NR
Zureikat 2016^8^	TR	NR	WP-67%; PP-33%	0%	NR	S	NR	R	WP-54%; PP-46%	0%	NR	S	NR	S

*TR* totally robotic; *H* hybrid; *WP* Whipple procedure; *PP* pylorus preserving; *MVR* multivisceral resection; *PJ* pancreaticojejunostomy; *PG* pancreaticogastrostomy; *DtM* duct-to-mucosa; *PJAS* pancreatojejunal anastomotic stent; *R* routinely; *S* selectively; *AC* antecolic; *RC* retrocolic; *NR* not reported.

^§^Studies that were excluded from the quantitative synthesis.

Intervention categories that were described for both open and robotic surgery included type of procedure (Whipple procedure, pylorus preserving PD, or multivisceral resection) and type of anastomosis (pancreaticojejunostomy, pancreaticogastrostomy and/or duct-to-mucosa). Vein resection was reported in 10 studies^8,28,30,35,36,38,41,42,46,47^ and was up to 25% in the robotic and up to 38% in the open group. Further categories described in Table 3 were routine or selective placement of pancreaticojejunal anastomotic stent, antecolic or retrocolic location of the jejunal loop, and routine or selective use of abdominal drainage.

### Meta-analysis

All 24 studies, regardless of the evidence level and risk of bias, were included in this meta-analysis. Primary outcomes were overall postoperative morbidity and margin involvement rate. Secondary clinical outcomes were operating time, estimated blood loss, postoperative pancreatic fistula (POPF), delayed gastric emptying (DGE), surgical site infection (SSI), reoperation rate, and length of hospital stay. An additional secondary outcome was the number of lymph nodes harvested.

#### Primary endpoints

##### Overall postoperative morbidity

**Figure 4:**
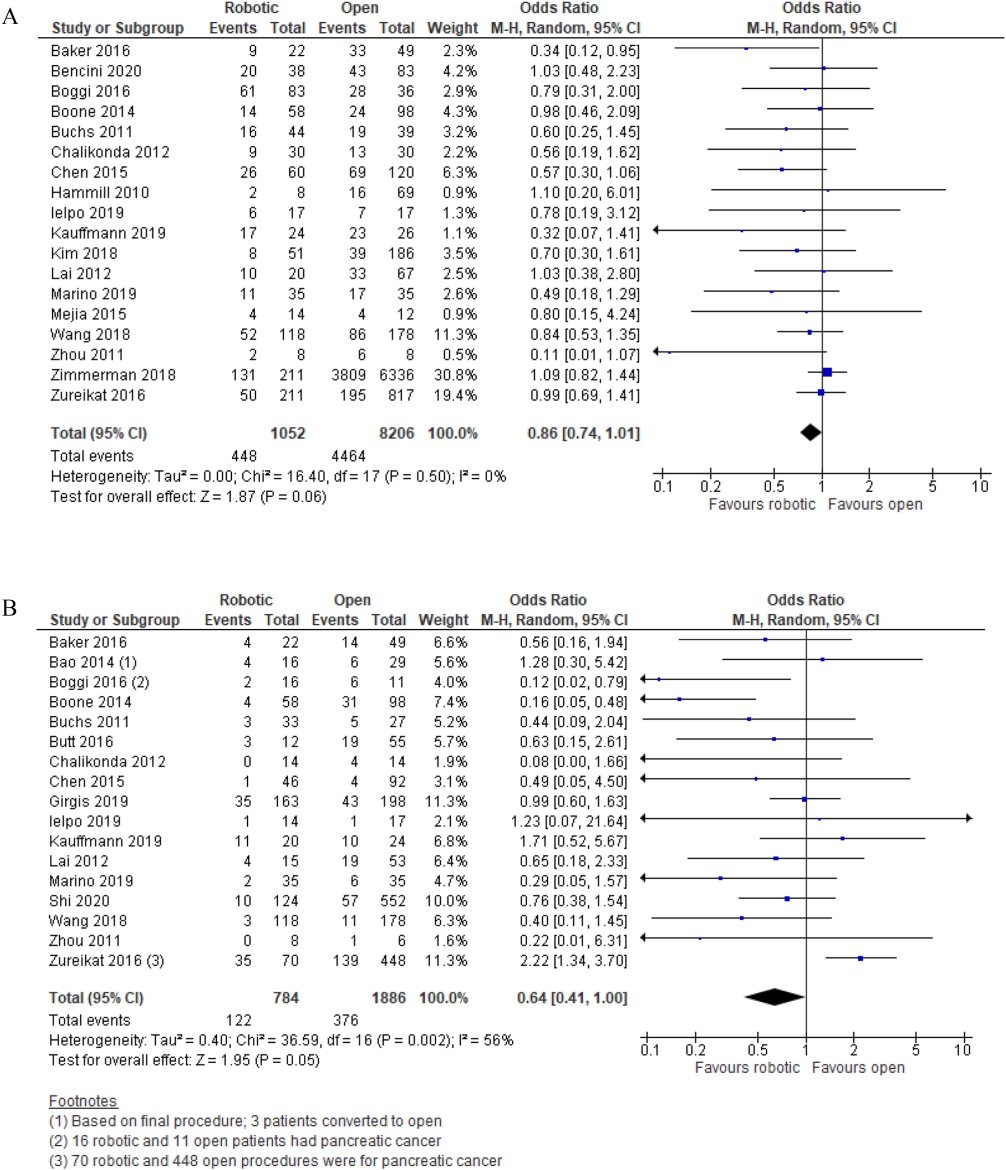
Meta-analysis of primary endpoints: (**A**) Overall postoperative morbidity. (**B**) Resection margin involvement rate.

Overall postoperative morbidity as a clinical primary outcome was reported in 18 studies (1052 robotic PD vs. 8206 open PD). The statistical among-study heterogeneity was low (I^2^ = 0%). The overall postoperative morbidity rate was 42.6% (448/1,052) in robotic PD vs. 54.4% (4,464/8,206) in open PD. This difference was not statistically significant [OR (95%CI) = 0.86 (0.74, 1.01); p = 0.06] (Fig. 4A). The RRR was 22% and the NNT was 9 (7, 12) (Table 4). 95% prediction interval was 0.72, 1.02 with moderate GRADE certainty of evidence (Table 4).

**Table 4:** Clinical relevance and certainty of evidence provided by statistical difference in clinical and pathologic endpoints (dichotomous and numeric) between robotic vs. open pancreaticoduodenectomy.

Dichotomous endpoints	RRR	ARR (95%CI)	NNT (95%CI)	95% prediction interval	GRADE certainty of evidence
Postoperative overall morbidity	0.22	0.118 (0.086, 0.149)	9 (7, 12)	0.72, 1.02	Moderate ⊕⊕⊕◯
POPF rate	0.13	0.021 (0.004, 0.038)	47 (26, 267)	0.29, 2.74	Low ⊕⊕◯◯
DGE rate	0.03	0.005 (− 0.017, 0.026)	210 (> 38 to benefit, > 4 to harm)	0.51, 1.87	Very low ⊕◯◯◯
Incisional SSI rate	0.22	0.022 (0.004, 0.040)	46 (25, 243)	0.12, 1.70	Low ⊕⊕◯◯
Reoperation rate	0.01	0.000 (− 0.013, 0.014)	3,007 (> 76 to harm, > 72 to benefit)	0.61, 1.04	Very low ⊕◯◯◯
Margin involvement rate	0.22	0.044 (0.013, 0.075)	23 (13, 79)	0.15, 2.68	Moderate ⊕⊕⊕◯

Numeric endpoints	MD (95% CI)	Clinical importance of the MD	95% prediction interval	GRADE certainty of evidence	
Operating time	75.17 (48.05, 102.28)	Moderate	− 58.77, 209.11	Low ⊕⊕◯◯	
Estimated blood loss	− 191.35 (− 238.12, − 144.59)	Low	− 382.04, − 0.66	Moderate ⊕⊕⊕◯	
Length of hospital stay	− 1.00 (− 1.88, − 0.12)	Moderate	− 4.32, 2.32	Very low ⊕◯◯◯	
Number of lymph nodes harvested	2.88 (1.12, 4.65)	Moderate	− 3.97, 9.73	Low ⊕⊕◯◯	

*RRR* relative risk reduction; *ARR* absolute risk reduction; *NNT* numbers needed to treat; *95%CI* 95% confidence interval; *POPF* postoperative pancreatic fistula; *DGE* delayed gastric emptying; *SSI* surgical site infection; *MD* mean difference.

##### Resection margin involvement rate

Resection margin involvement rate was reported in 17 studies (784 robotic PD vs. 1886 open PD). The statistical among-study heterogeneity was moderate (I^2^ = 56%; Tau^2^ = 0.40). The margin involvement rate was 15.6% (122/784) in robotic PD vs. 19.9% (376/1886) in open PD. This difference was statistically and clinically significant [OR (95%CI) = 0.64 (0.41, 1.00); p = 0.05; NNT = 23 (13, 79)] (Fig. 4B) (Table 4). 95% prediction interval was 0.15, 2.68 with moderate GRADE certainty of evidence (Table 4).

#### Secondary endpoints

##### Operating time

**Figure 5:**
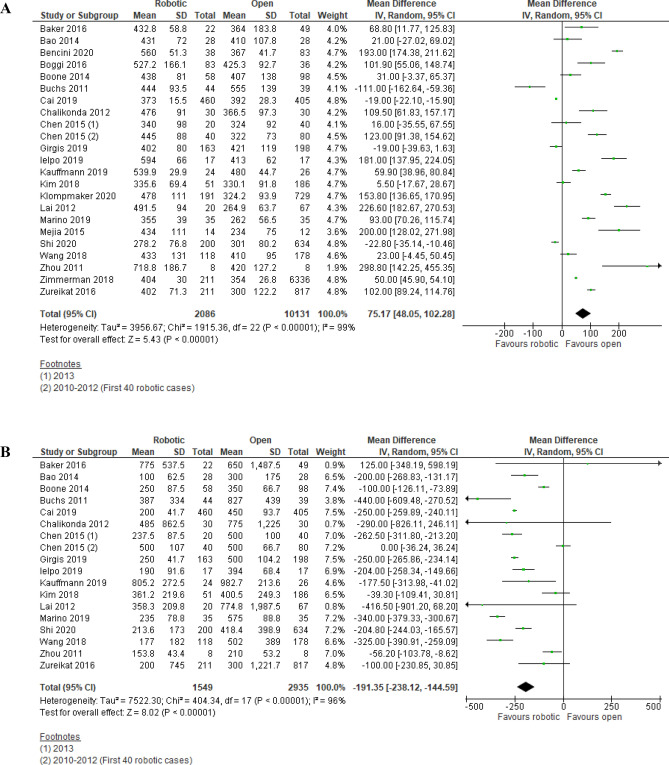

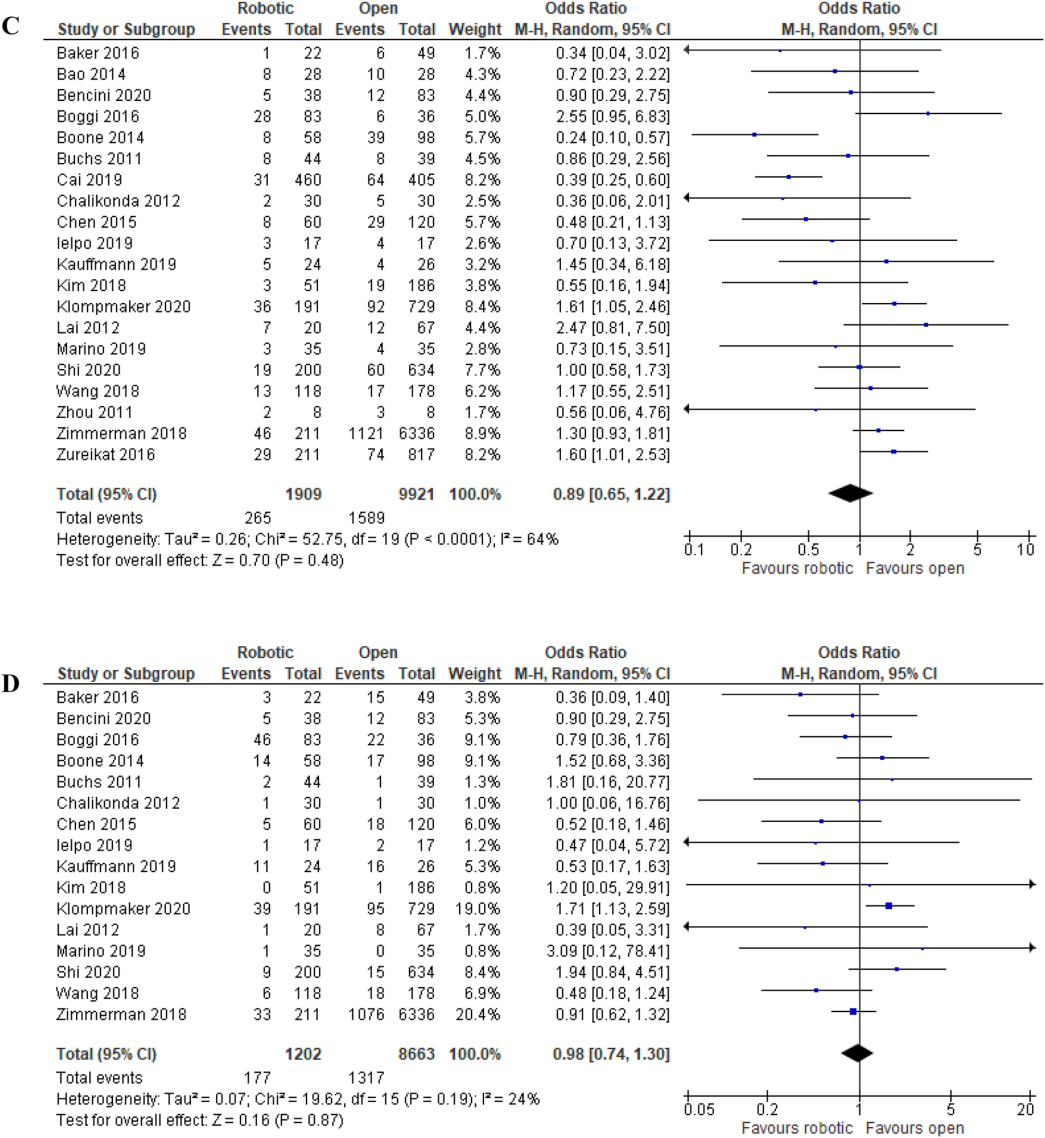

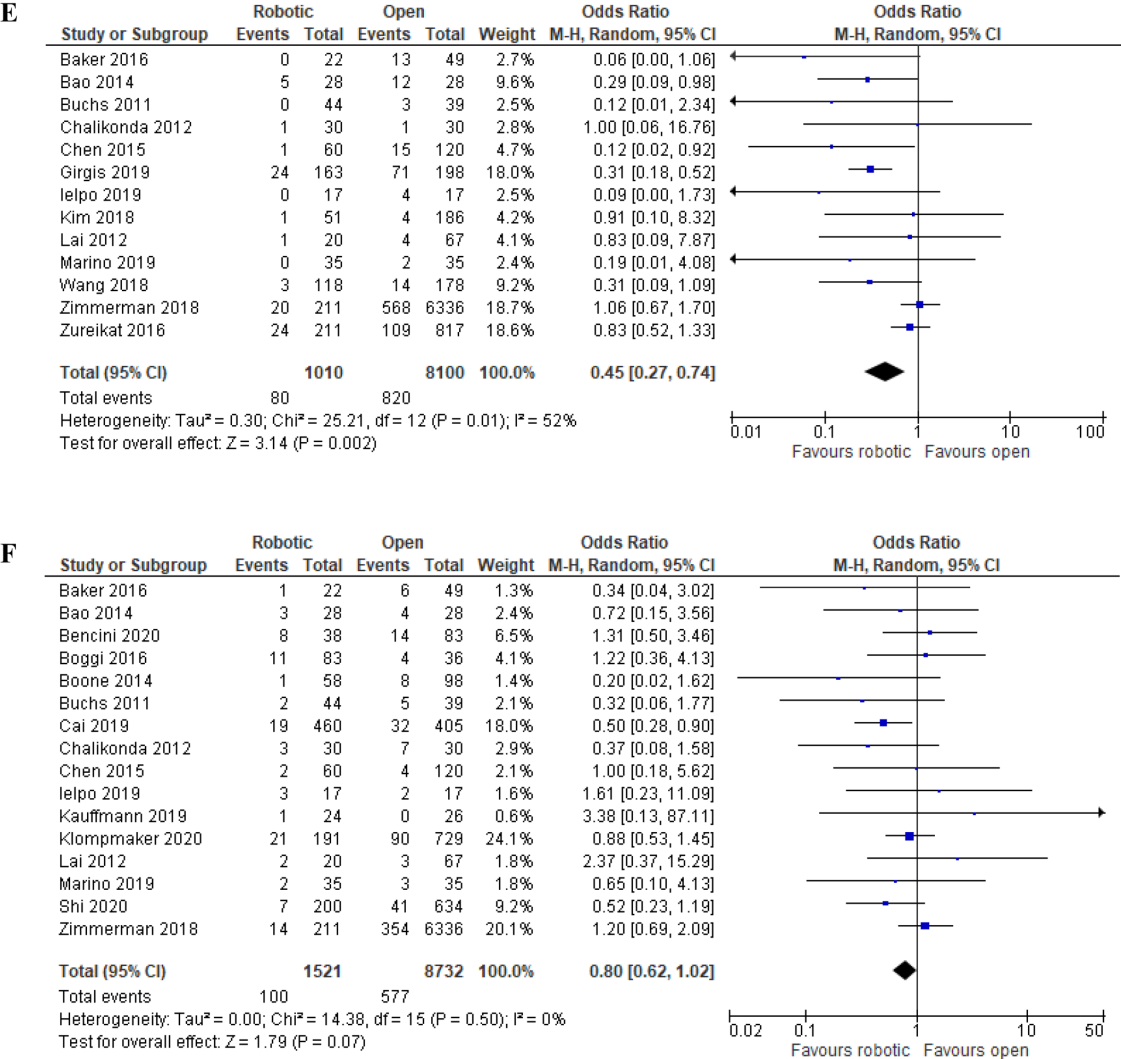

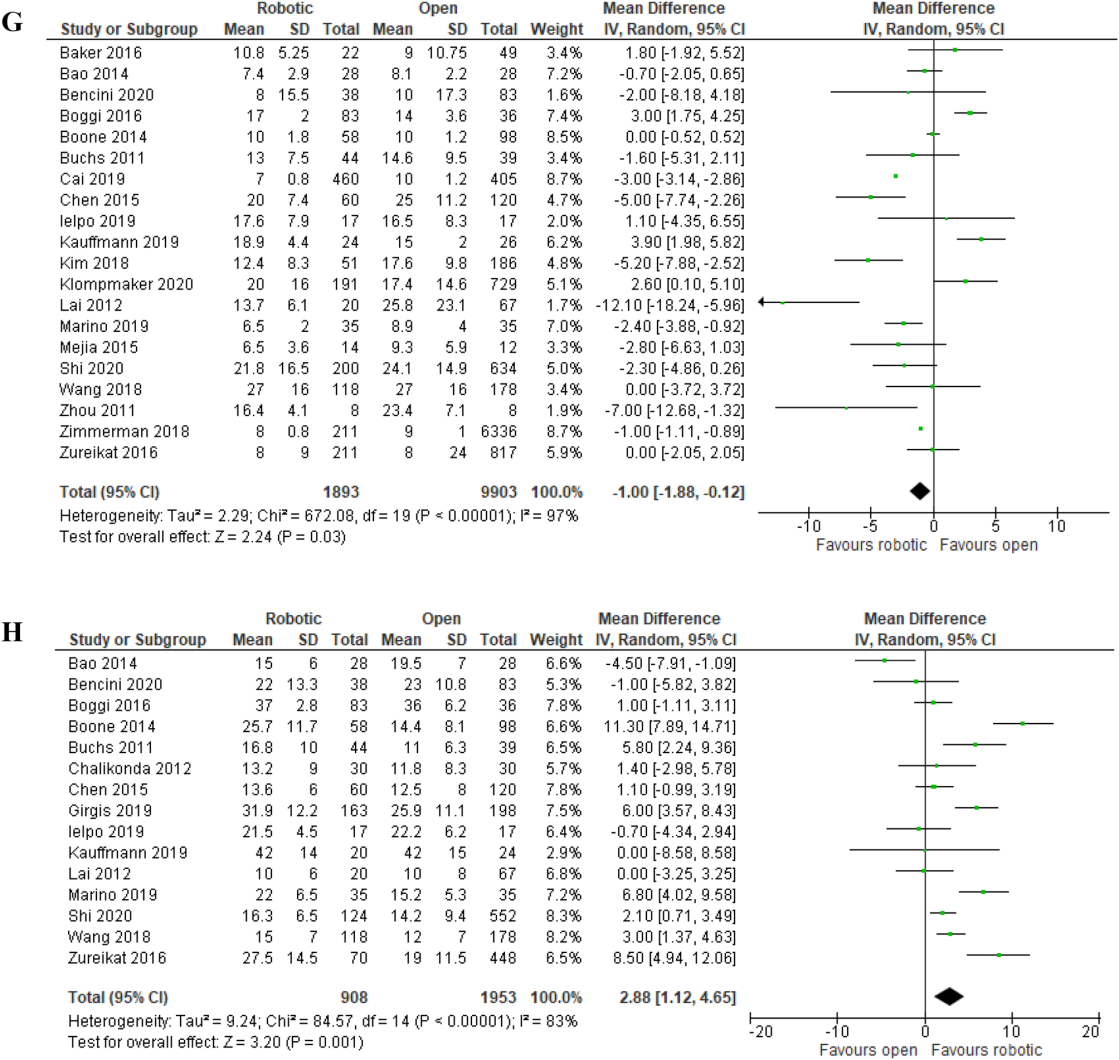
Meta-analysis of secondary endpoints: (**A**) Operating time. (**B**) Estimated blood loss. (**C**) Postoperative pancreatic fistula rate. (**D**) Delayed gastric emptying rate. (**E**) Incisional SSI rate. (**F**) Reoperation rate. (**G**) Length of hospital stay. (**H**) Number of lymph nodes harvested.

Operation time was reported in 23 studies (2,086 robotic PD vs. 10,131 open PD) and was significantly longer in robotic PD [MD (95%CI) = 75.17 (48.05, 102.28); p < 0.00001] with high among-study statistical heterogeneity (I^2^ = 99%; Tau^2^ = 3956.67) (Fig. 5A). Although the clinical importance of the MD was assessed to be moderate, 95% prediction interval was − 58.77, 209.11 and GRADE certainty of evidence was low (Table 4).

##### Estimated blood loss

Estimated blood loss was reported in 18 studies (1,549 robotic PD vs. 2,935 open PD) and was significantly lower in robotic PD [MD (95%CI) = − 191.35 (− 238.12, − 144.59); p < 0.00001] with high among-study statistical heterogeneity (I^2^ = 96%; Tau^2^ = 7,522.30) (Fig. 5B). Clinical importance of the MD was assessed to be low and 95% prediction interval was − 382.04, − 0.66 with moderate GRADE certainty of evidence (Table 4).

##### Postoperative pancreatic fistula rate

POPF rate was reported in 20 studies and did not differ significantly between the two approaches (18.9% (265/1,909) in robotic PD vs. 16.0% (1,589/9,921) in open PD) [OR (95%CI) = 0.89 (0.65, 1.22); p = 0.48; NNT = 47 (26, 267)] with high among-study heterogeneity (I^2^ = 64%; Tau^2^ = 0.26) (Fig. 5C) (Table 4). 95% prediction interval was 0.29, 2.74 with low GRADE certainty of evidence (Table 4).

##### Delayed gastric emptying rate

Sixteen studies reported the rate of delayed gastric emptying, which did not differ significantly between the two approaches (14.7% (177/1,202) in robotic PD vs. 15.2% (1,317/8,663) in open PD) [OR (95%CI) = 0.98 (0.74, 1.30); p = 0.87], with low among-study heterogeneity (I^2^ = 24%; Tau^2^ = 0.07) (Fig. 5D). RRR was 3%, NNT was 210, 95% prediction interval was 0.51, 1.87 with very low GRADE certainty of evidence (Table 4).

##### Incisional surgical site infection rate

Incisional SSI rate was reported in 13 studies and favored robotic PD (7.9% (80/1,010) in robotic PD vs. 10.1% (820/8,100) in open PD) [OR (95%CI) = 0.45 (0.27, 74); p = 0.002; NNT = 46 (25, 243)] with moderate among-study heterogeneity (I^2^ = 52%; Tau^2^ = 0.30) (Fig. 5E) (Table 4). 95% prediction interval was 0.12, 1.70 with low GRADE certainty of evidence (Table 4).

##### Reoperation rate

Sixteen studies reported the rate of reoperations (1,521 robotic PD vs. 8,732 open PD), which did not differ significantly between the two approaches (6.6% (100/1,521) in robotic PD vs. 6.6% (577/8,732) in open PD) [OR (95%CI) = 0.80 (0.62, 102); p = 0.72], with low among-study heterogeneity (I^2^ = 0%; Tau^2^ = 0.00) (Fig. 5F). RRR was 1%, NNT was 3,007, 95% prediction interval was 0.61, 1.04 with very low GRADE certainty of evidence (Table 4).

##### Length of hospital stay

Length of hospital stay was reported in 20 studies (1,893 robotic PD vs. 9,903 open PD) and did not differ significantly between the two approaches [MD (95%CI) = − 1.00 (− 1.88, − 0.12); (p = 0.06)] with high among-study heterogeneity (I^2^ = 97%; Tau^2^ = 2.29) (Fig. 5G). Although the clinical importance of the MD was assessed to be moderate, 95% prediction interval was − 4.32, 2.32 and GRADE certainty of evidence was very low (Table 4).

##### Number of lymph nodes harvested

Number of lymph nodes harvested was reported in 15 studies (908 robotic PD vs. 1,953 open PD). Statistical among-study heterogeneity was high (I^2^ = 83%; Tau^2^ = 9.24). The difference was statistically significant [MD (95%CI) = 2.88 (1.12, 4.65); p = 0.001] (Fig. 5H). Although the clinical importance of the MD was be moderate, 95% prediction interval was − 3.97, 9.73 and GRADE certainty of evidence was low (Table 4).

#### Meta-regression analysis

**Figure 6:**
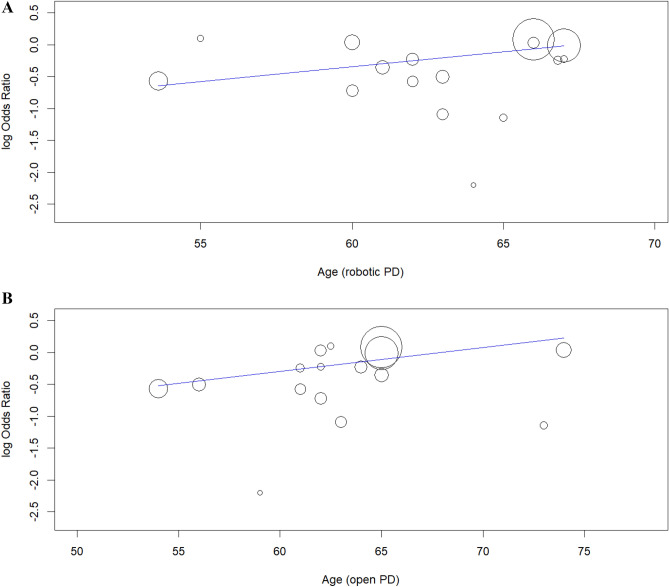
Ad-hoc meta-regression analysis: (**A**) Regression plot of overall postoperative morbidity and average age in robotic PD (Omnibus p = 0.040). (**B**) Regression plot of overall postoperative morbidity and average age in open PD (Omnibus p = 0.075).

Ad-hoc meta-regression analysis was performed to assess the impact of potential covariates on the statistical findings. Covariates utilized for meta-regression analysis included the central tendency values for age and BMI, proportion of males, proportion patients with ASA > 2, and study design. A statistically significant correlation was found between overall postoperative mortality and average age in robotic PD (Omnibus p = 0.040) (Fig. 6A). However, only a statistical trend in correlation was found between overall postoperative mortality and open PD (Omnibus p = 0.075) (Fig. 6B). No statistically significant impact of the above-mentioned covariates on margin involvement rate and secondary endpoints was found.

#### Publication bias and sensitivity analysis

**Figure 7:**
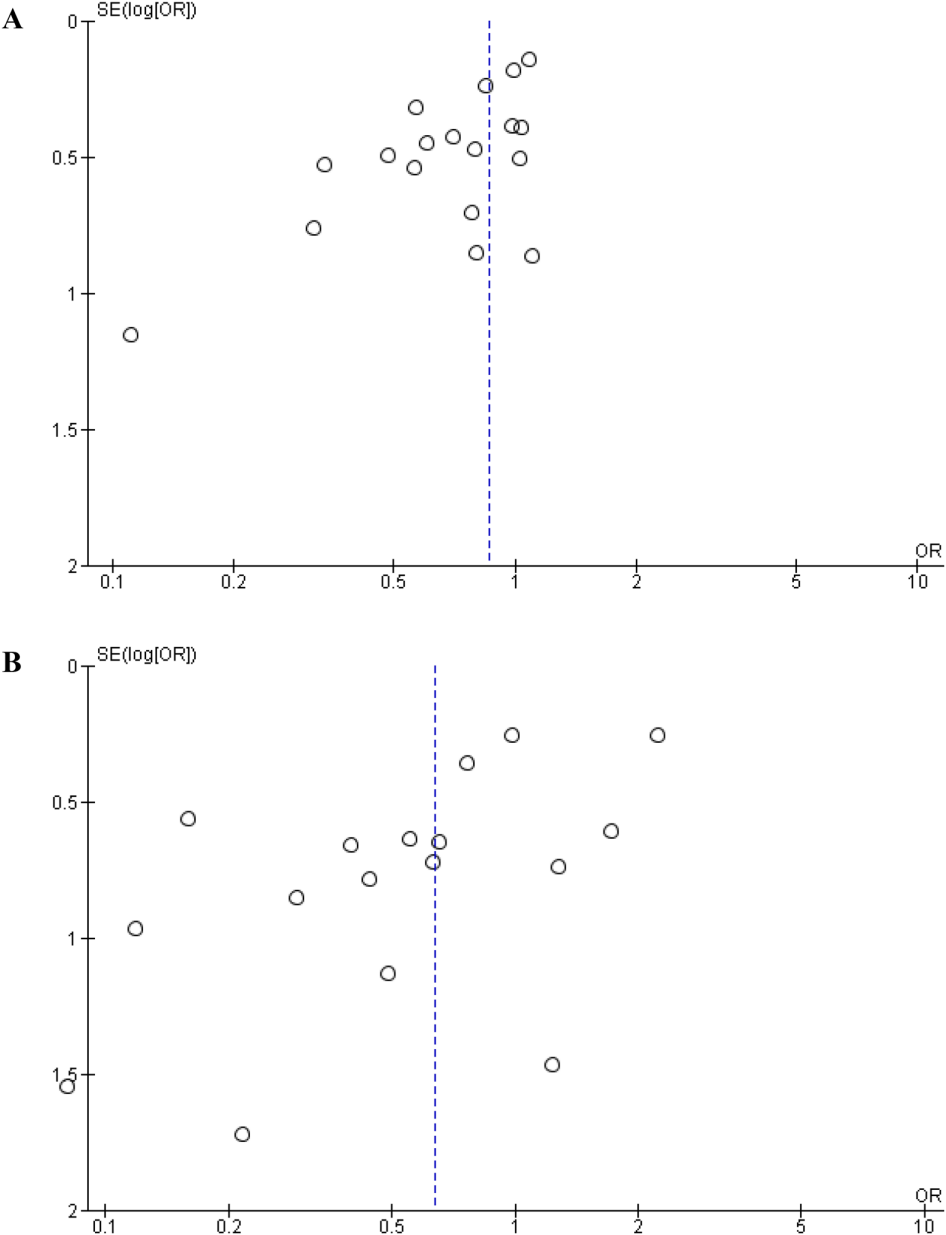
Funnel plot of reporting bias: (**A**) Overall postoperative morbidity. (**B**) Resection margin involvement rate.

**Figure 8:**
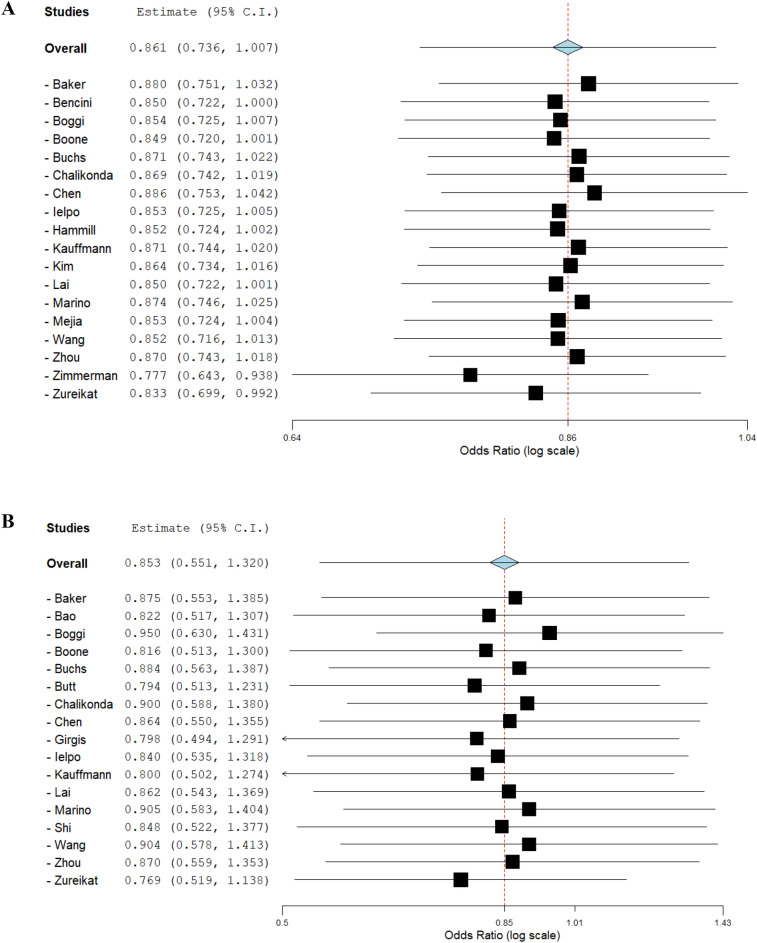
Sensitivity analysis: (**A**) Leave-one-out meta-analysis of overall postoperative morbidity. (**B**) Leave-one-out meta-analysis of margin involvement rate.

Publication bias was evaluated by visual assessment of symmetry on the funnel plot (Fig. 7) and using Egger’s test (Overall postoperative morbidity: t = 0.534, p = 0.522; Margin involvement rate: t = 0.478, p = 0.641). No significant risk of publication bias was found. A sensitivity analysis of the included observational studies was performed using leave-one-out forest plots. Consecutive exclusion of studies did not significantly impact the findings (Fig. 8). The results of the evaluation of the certainty of evidence are summarized in Table 4.

## Discussion

Currently, general surgery is the fastest growing specialty for the DaVinci robotic platform in the US. Specifically, robotic PD is experiencing widespread growth since its introduction^7^. Similar to laparoscopic distal pancreatectomy has seen its use expanded three-fold between 1998 and 2009, robotic PD is also subject to significant growth nationwide^56^.

Pancreaticoduodenectomy remains a technically demanding operation with significant risks of morbidity^57^. Historically, minimally invasive surgery has often been compared to open approach in non-inferiority studies. With improved visualization through magnification of target anatomy and ergonomics allowing more precise excision along critical resection margins, robotic approach may allow superior dissection and skeletonization of critical borders. Margins near the uncinate process along the superior mesenteric artery requiring dissection down to the adventitia is facilitated with the robotic instruments. Although resection margins are important for overall survival and locoregional recurrence, the ability to achieve R0 resection can be as low as 60% in some open cases^14,58^. One of the important findings of this meta-analysis is the improvement in resection margin in robotic PD. Patients with non-involved resection margins have improved overall survival as well as decreased locoregional recurrence risk in comparison to R1 resection^14^. The benefits of R0 resection is especially pronounced in patients with N0 disease^14^.

Previously, the benefits of robotic surgery for pancreatic cancer in terms of margin status have been reported^12,45^. With the high rate of locoregional failure, assessment of the circumferential margin of the Whipple specimen was re-defined in 2006^58^. Verbeke et al. advocated a standardized protocol for margin assessment since circumferential margin positivity can be underestimated by as much as 60%^59^. Unfortunately, the method of margin quantification in the majority of these studies were not clearly defined based on the papers reviewed^58^. Furthermore, only two studies stated their adherence to the standardized Leeds Pathology Protocol (LEEPP) for margin assessment. Only two papers^45,49^ specified that the LEEPP protocol were followed. Nonetheless, Peng et al. performed a meta-analysis previously and showed improved margin status favoring robotic surgery over open surgery^12,60^. Within their findings, only 8 studies were included which discussed oncologic outcomes^12^. Kauffman et al. performed a propensity score matched analysis of robotic versus open PD and found equivalent rates of R1 resection^45^. The authors did comment, as speculated by many robotic surgeons, that following the peri-adventitial dissection plane close to the right side of the SMA, following early ligation of the inferior pancreaticoduodenal artery makes the retroperitoneal dissection easier. The retroperitoneal dissection plan is particularly efficient using the minimally invasive robotic approach^45^. In this meta-analysis, we confirmed the significant difference in resection margin involvement rates favoring robotic approach.

Similar to margin status, an increase in the number of lymph nodes harvested is frequently associated with improved staging and optimal resection margins^17^. In this study, we also identified that robotic PD has an increased number of lymph node harvested as compared to open. Previously, studies have shown both that total number of lymph nodes evaluated and a higher positive lymph node ratio to be superior in terms of oncological outcome. This meta-analysis is one of the first to present superior nodal sampling with the use of robotic surgery.

As expected, clinical outcomes favoring robotic surgery included significantly lower estimated blood loss, decreased incisional SSI rate, and lower length of hospital stay at the cost of longer operating time. These findings confirmed the results of previous meta-analyses. No significant difference in POPF, DGE, and reoperation rates was found.

One of the strengths of this meta-analysis is the number of studies and thereby number of patients included. Other strengths were prospective development and registration of the protocol, and rigorous literature search. This meta-analysis has several limitations. Given the observational nature, all included studies were subject to high risk of selection, performance, and detection biases. Moreover, all studies reported only short-term outcomes. The differences in surgical approaches and perioperative management across the globe may have contributed further to the heterogeneity and variance across the included studies. The lack of other histopathological details including and not limited to lymphovascular and perineural invasion adds additional heterogeneity. Another limitation was a lack of standardization in the definitions of interventions, a fact that may have contributed to the risk of performance bias.

## Conclusion

This meta-analysis found that robotic PD was associated with improved resection margins and number of lymph node harvested as compared to open PD. Moreover, robotic PD allowed surgery with less blood loss and was associated with decreased wound infection rates and shorter length of hospital stay, at the expense of increased operating time and surgical cost. The current application of robotic PD needs further experimental and observational prospective studies given the possible benefits over open PD.

## Supplementary Information

Supplementary Information.

## Data Availability

The datasets generated during and/or analyzed during the current study are available from the corresponding author on reasonable request.
