# Supplementary material for: Robotic pancreaticoduodenectomy provides better histopathological outcomes as compared to its open counterpart: a meta-analysis
Source: Sci Rep. 2021 Feb 12;11:3774. doi: 10.1038/s41598-021-83391-x (PMC7881190; doi:10.1038/s41598-021-83391-x)
Supplement: Supplementary file 1 — Supplementary Information. [file 41598_2021_83391_MOESM1_ESM.pdf]

Robotic pancreaticoduodenectomy provides better histopathological outcomes as compared to its open counterpart: A meta-analysis.

Xiang Da (Eric) Dong [1*]; Daniel Moritz Felsenreich [2]; Shekhar Gogna [1]; Aram Rojas [1]; Ethan Zhang [1]; Michael Dong [1]; Asad Azim [1]; Mahir Gachabayov [1*]

1 Department of Surgery, Westchester Medical Center, New York Medical College, Valhalla, NY, USA

2 Division of General Surgery, Department of Surgery, Vienna Medical University, Vienna, Austria

Supplement 1. Pubmed search strategy.

("pancreatoduodenectomy"[Title/Abstract] OR "pancreaticoduodenectomy"[Title/Abstract] OR "whipple"[Title/Abstract]) AND "robotic"[Title/Abstract]

Supplement 2. Quality assessment of included observational studies according to Risk Of Bias In Non-randomized Studies (ROBINS-I) tool.

Study	Bias due to confounding	Bias in selection of participants into the study	Bias in the classification of intervention	Bias due to deviations from intended interventions	Bias due to missing data	Bias in the measurement of outcomes	Bias in selection of reported result	Overall RoB
Baker 2016	Moderate	Serious	Low	Low	Low	Low	Moderate	Moderate
Bao 2014	Serious	Serious	Low	Low	Low	Low	Moderate	Serious
Bencini 2020	Moderate	Serious	Low	Low	Moderate	Moderate	Serious	Serious
Boggi 2016	Serious	Serious	Low	Low	Low	Low	Moderate	Moderate
Boone 2014	Serious	Serious	Serious	Low	Serious	Moderate	Serious	Serious
Buchs 2011	Serious	Serious	Low	Low	Low	Low	Moderate	Moderate
Butt 2016	Serious	Serious	Serious	Low	Serious	Moderate	Serious	Serious
Cai 2019	Serious	Serious	Low	Low	Moderate	Moderate	Serious	Serious
Chalikonda 2012	Moderate	Serious	Low	Low	Low	Low	Moderate	Moderate
Chen 2015	Moderate	Serious	Low	Low	Low	Low	Moderate	Moderate
Girgis 2019	Serious	Serious	Moderate	Low	Low	Low	Moderate	Serious
Ielpo 2019	Serious	Serious	Moderate	Low	Low	Low	Moderate	Serious
Hammill 2010	Serious	Serious	Serious	Low	Serious	Moderate	Serious	Serious
Kauffmann 2019	Serious	Serious	Moderate	Low	Low	Low	Moderate	Serious
Kim 2018	Serious	Serious	Low	Low	Moderate	Moderate	Serious	Serious
Klompmaker 2020	Serious	Serious	Low	Low	Moderate	Moderate	Serious	Serious
Lai 2012	Serious	Serious	Low	Low	Low	Low	Moderate	Serious
Marino 2019	Serious	Serious	Moderate	Low	Low	Low	Moderate	Serious
McMillan 2017	Serious	Serious	Moderate	Low	Moderate	Moderate	Serious	Serious
Mejia 2015	Serious	Serious	Serious	Low	Serious	Moderate	Serious	Serious
Napoli 2018	Serious	Serious	Low	Low	Moderate	Moderate	Serious	Serious
Shi 2020	Serious	Serious	Low	Low	Low	Low	Moderate	Serious
Varley 2019	Serious	Serious	Serious	Low	Moderate	Moderate	Serious	Serious
Walsh 2011	Serious	Serious	Serious	Low	Serious	Moderate	Serious	Moderate
Wang 2018	Moderate	Serious	Low	Low	Low	Low	Moderate	Moderate
Wilson 2019	Serious	Serious	Serious	Low	Serious	Moderate	Serious	Serious
Zhou 2011	Serious	Serious	Moderate	Low	Low	Low	Moderate	Serious
Zimmerman 2018	Serious	Serious	Serious	Low	Moderate	Moderate	Serious	Serious
Zureikat 2016	Serious	Serious	Low	Low	Low	Low	Moderate	Moderate
